# The relationship between the degeneration and asymmetry of the lumbar multifidus and erector spinae muscles in patients with lumbar disc herniation with and without root compression

**DOI:** 10.1186/s13018-022-03444-3

**Published:** 2022-12-14

**Authors:** Alikemal Yazici, Tuba Yerlikaya

**Affiliations:** 1 Faculty of Medicine, Orthopaedics and Traumatology Department, Near East University, Nicosia, Cyprus; 2 Orthopaedics and Traumatology Department, Buyuk Anadolu Hospital, Samsun, Turkey; 3 Faculty of Health Sciences, Physiotherapy and Rehabilitation Department, Near East University, Nicosia, Cyprus

**Keywords:** Lumbar disc herniation, Root compression, Paraspinal muscle degeneration, Asymmetry, Magnetic resonance imaging

## Abstract

**Background:**

The determination of muscle pathologies in lumbar disc herniation (LDH) and other conditions with low back pain is important for understanding low back problems and determining appropriate treatment methods. In patients with lumbar disc herniation with radiculopathy, elucidating the effect of root compression on the severity of muscle degeneration may predict the importance of alleviating root compression. For this purpose, magnetic resonance imaging (MRI) was used to compare the degeneration and asymmetries of the lumbar musculus multifidus (MF) and lumbar musculus erector spinae (ES) muscles in patients with lumbar discopathy without root compression (radiculopathy) and in patients with lumbar discopathy with root compression (radiculopathy).

**Methods:**

The patients were examined in two groups: 56 patients with lumbar discopathy and no radiculopathy (Non-rad group) and 51 patients with lumbar discopathy and radiculopathy (Rad group). On axial MRI sections passing through the centre of the disc at the L3-S1 level, the asymmetry, cross-sectional area (CSA), fat infiltration, and total CSA (TCSA = MF + ES) of the MF and ES muscles were measured and compared.

**Results:**

No difference was seen between the groups with respect to the CSA values of the right and left MF and left ES, but a significant difference was found in the right ES CSA (*p* = 0.021). The CSA and TCSA of the MF and ES showed no asymmetry according to group. Severe fat infiltration of > 50% in the right and left MF and left ES was found in the Rad group at a higher rate than in the Non-rad group. Fat infiltration was significantly positively correlated with age, body mass index, and the duration of pain (*p* < 0.001, *p* < 0.001, *p* = 0.004, respectively).

**Conclusions:**

The study results showed a correlation between LDH and paraspinal muscle degeneration, while no correlation was found with asymmetry. Severe (> 50%) fat infiltration is associated with root compression, and the severity of fat filtration increases in the presence of root compression. The development of more severe degeneration due to denervation associated with root compression plays a role in the emergence of this situation. Therefore, in patients with lumbar disc herniation with radiculopathy, it can be foreseen that to stop and correct severe fat infiltration and muscle degeneration, first, nerve root compression should be corrected with appropriate medical treatment methods, and in patients in whom there is no response, the pressure should be alleviated with appropriate surgical methods.

## Background

Lumbar disc herniation (LDH) is one of the causes of low back pain. Asymptomatic LDH is seen at a rate of 0.5–20%, and symptomatic LDH is seen at a rate of 1–3%. The male/female ratio of LDH is 2/1, and LDH is most often seen in patients between 30 and 50 years of age. In 95% of patients, LDH is at the level of L4-5 or L5-S1 [1–4]. The localised or widespread migration of disc material (nucleus pulposus or annulus fibrosis) to outside the intervertebral disc space is referred to as LDH [1, 5]. Symptoms and clinical findings can be seen as pain, muscle spasm, restricted movement, and sensory and motor neurological deficits depending on the size and level of disc herniation [6, 7]. In clinical practice, LDH is said to affect the paraspinal muscles, which are generally overlooked [7, 8].

The paraspinal muscles are responsible for the control of intersegmentary movements with movements of the spine and extremities, and they have unique architecture and design features [9, 10]. The MF and ES from the paraspinal muscles form the two main muscle groups that function in lumbar stabilisation and mobilisation [9, 11, 12]. In addition to these two muscles, the psoas muscle is appropriately placed to help stabilise the lumbar cylinder, in which the upper section is the diaphragm, the lower section is the pelvic floor, and the middle is the transversus abdominis muscle [13–15]. The psoas is referred to as a rod providing rigidity to and the stabilisation of the lumbar spine in the absence of posterior support [16]. In addition, the psoas contributes to anterior pelvic tilt balance and the rigidity of the lumbar spine as a compensatory mechanism [17].

The paraspinal muscles are of critical importance for spinal health. Paraspinal muscle dysfunction and failure are common in patients with low back pain [18]. Many spinal pathologies (LDH and other causes of low back pain) cause changes in the paraspinal muscles. These changes include an increase in fat and connective tissue in the muscle, fibre type changes, distorted cell populations, altered gene expression, low muscle resistance, low muscle strength, and muscle atrophy [19–26]. These findings are seen in parallel with the denervation and reinnervation of the paraspinal muscles in disc herniation and nerve root compression [18, 27].

Many studies have been conducted to reveal the status of the paraspinal muscles in patients with LDH and other causes of low back pain. Changes in muscle tissue composition, such as fat-fibrotic infiltration (as fatty tissue is not contractile, fat infiltration deteriorates the quality of the paraspinal muscles) [28, 29], muscle atrophy, and changes in fibre types, have been shown to be different at the point of asymmetry in patient populations and animal studies compared with control groups. Some of these studies have been clinical observational studies [26, 30–37], some have been histological studies [19, 38], and some have examined stem cells and performed gene analyses [22, 23, 39]. There are also publications in the literature of experimental animal model studies [24, 27, 40–43] and studies that have examined the changes occurring naturally in animals [44, 45].

Significant histological differences have been shown between the normal and disc hernia sides [27, 46, 47]. Previous studies have reported that a higher rate of fat-fibrotic infiltration is observed in the paraspinal muscles in patients with LDH; type I fibres (slow twitch and fatigue-resistant fibres) decrease, and type IIX fibres (fast twitch fibres less resistant to fatigue) increase. In addition, it has been shown that paraspinal muscles contain more stem cells in patients with LDH, and these stem cells have higher fibrogenic and adipogenic gene expression [19, 35, 48].

Studies examining the relationships between paraspinal muscle degeneration and dysfunction [23–27, 30, 34, 49–54] and pain in the structures in the spinal region (skin, fat tissue, fascia, ligaments, muscles) [55] could be of guidance in the prevention or treatment of spinal pathologies. Thus, the primary aim of this study was to compare the relationships between the CSA and fat infiltration of the MF and ES muscles in patients with low back pain with and without nerve root compression associated with LDH.

The secondary aims of the study were

- To reveal the relationship between muscle asymmetry and degeneration and the severity of radiculopathy,
- To reveal whether root compression affects the severity of muscle degeneration by determining the relationships among demographic/physical characteristics, segmentation and herniation.

Thus, the results obtained may guide clinicians in their application of conservative and/or surgical treatment.

## Methods

### Participants

This observational cross-sectional study included 107 patients who visited the Orthopaedics and Traumatology Clinic between February and May 2021 with complaints of low back pain and were diagnosed with LDH through magnetic resonance imaging (MRI). The patients were aged 20–65 years and described ongoing low back pain for the last 3 months. Two groups were formed of patients with low back pain with radiculopathy (Rad group) and without radiculopathy (Non-rad group). All the study participants signed informed consent forms. Approval for the study was granted by the Local Ethics Committee (YDU/2021/87-1273).

The Non-rad group included 56 patients with LDH but no degenerative disc changes (degenerative disc disease) or vertebral endplate changes (Modic changes) on MRI, no findings of nerve root compression in the neuromuscular examination, no radicular pain, and negativity in specific lumbar tests (straight leg raise, contralateral straight leg raise, Lasegue’s test, femoral nerve tension test). When nerve compression was suspected, EMG was requested. Patients with nerve compression determined on MRI and EMG and those with degenerative disc disorders were not included in the Non-rad group. The Rad group included 51 patients with radicular pain in the leg, positivity in the specific lumbar tests, and findings of nerve root compression on EMG and MRI.

MRI was performed to reveal the aetiology of the low back pain. The hernia types were classified as median, paramedian, foraminal, or extraforaminal according to the location of the protruding section of the disc and as bulging, protrusion, extrusion, or sequestration based on the degree of protrusion on MRI [38, 39]. The MRI-based nerve compression grading system was used to classify radiculopathy in this study [51, 52].

Low back pain was defined as pain between the inferior edge of the costae and the gluteal fold. Radicular pain was defined as pain radiating from below the gluteal fold to the thigh, leg, and foot. The pain was described by the patient and confirmed by the physician performing the examination. Pain intensity was evaluated using a standard 100 mm visual analogue scale (VAS).

Patients were excluded from the study if they had deformities (scoliosis, etc.) affecting the spine, malformations, pelvic disorders, limb length discrepancies, additional coxarthrosis, rheumatological diseases (rheumatoid arthritis, ankylosing spondylitis), infections, a history of lumbar surgery and/or low back treatment, vertebral fractures, spondylolisthesis, epidural injections, radiofrequency ablations, metabolic diseases (diabetes mellitus, etc.), obesity, pregnancy, or a history of malignancy.

Demographic, clinical, and disease-related data were obtained from the patients in face-to-face interviews. In patients where a differential diagnosis was necessary, C-reactive protein (CRP), antistreptolysin O (ASO), rheumatoid factor (RF), haemogram, erythrocyte sedimentation rate (ESR), and Salmonella and Brucella tests, as well as a full urine analysis, were requested. The examinations of the 107 patients were performed by two physicians with 25 years of experience in spinal surgery, and the lumbar spine MR images were analysed by an experienced consultant and radiology specialist blinded to the clinical history of the patients. The MR images were all taken by the same radiology technician.

## Measurements

### Magnetic resonance imaging

Imaging was performed with a 1.5 Tesla MR device (Signa Explorer SV25.3 16 channel, up-to-date software, General Electric, Milwaukee, WI, USA). The images were obtained with the patient in a supine position, with a routine protocol directed to the lumbar spine and with the measurement level between L3 and S1 (L3-4/L4-5/L5-S1) to be able to view the centre of the disc, parallel to the vertebral endplates. Turbo spin‒echo T1- and T2-weighted sagittal and turbo spin‒echo T2 axial 4-mm sections parallel to the disc spaces were taken. Evaluations were made on T2 axial sections. The asymmetry, fat content, CSA, and TCSA of the right and left sides of the MF and ES (m. iliocostalis and m. longissimus) were examined at the L3-S1 level. The technical values were TR 400–600, TE 15–25, thickness 4 mm, band width 25–150, and rotational angle 90° for T1 imaging and TR 3000–4000, TE 100–120, thickness 4 mm, band width 25–150, and rotational angle 130°–150° for T2 imaging, and the acquisition time was a mean of 10 min.

**Fig. 1:**
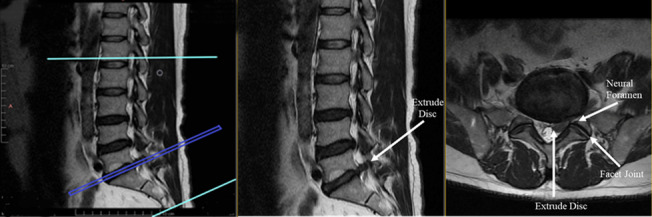
T2 sagittal and axial sections of L5-S1 of a patient in the radiculopathy group, showing more evident narrowing of both neural foramens on the left side; an indentation in the anterior epidural space, with fat obliterating the left lateral recess; contact with the S1 nerve root; and the appearance of an extruding disc

Assured reliability has been found in the evaluation of muscle-related changes on MRI [53, 54]. MRI has higher image resolution and provides better determination of muscle and soft tissues such as fat than ultrasonography and computed tomography [56–58]. Muscle degeneration is usually evaluated in axial MRI with T1- and T2-weighted sequences (Fig. 1) [54, 56, 58]. It has been reported that muscle degeneration can be observed on MRI scans as a decrease in muscle size or an increase in fat accumulation [20, 25].

In the objective evaluation of disc herniations, MRI and EMG are widely used methods. In the determination of disc diseases, MRI has become the gold standard. However, information about the functional and physiological status of neural and muscle tissues can be provided with the EMG method [59–61].

The indications for spinal MRI include degenerative disc disease and disc herniations and the evaluation of spinal deformities (scoliosis, kyphosis), spinal trauma, spinal instabilities, spinal stenosis, spinal infections, results of disc herniation surgery and instrumentation, implantation-related complications, and suspected cancer (primary and metastatic tumours of the spine) [62–64].

Generally, EMG is used in the diagnosis and differential diagnosis of peripheral neuropathy; motor neuron diseases; primary muscle diseases; and local (entrapment neuropathies), plexus (plexus brachialis), and radicular lesions. EMG has an important place in the differential diagnosis of radiculopathy in disc herniation and in the follow-up of prognosis [59–61, 65, 66]. Lower extremity sensory and motor nerve conduction tests were performed with EMG in the current study patients. The presence of denervation findings was evaluated with needle EMG in the muscles with L3, L4, L5, and S1 root innervation in both lower extremities.

**Fig. 2:**
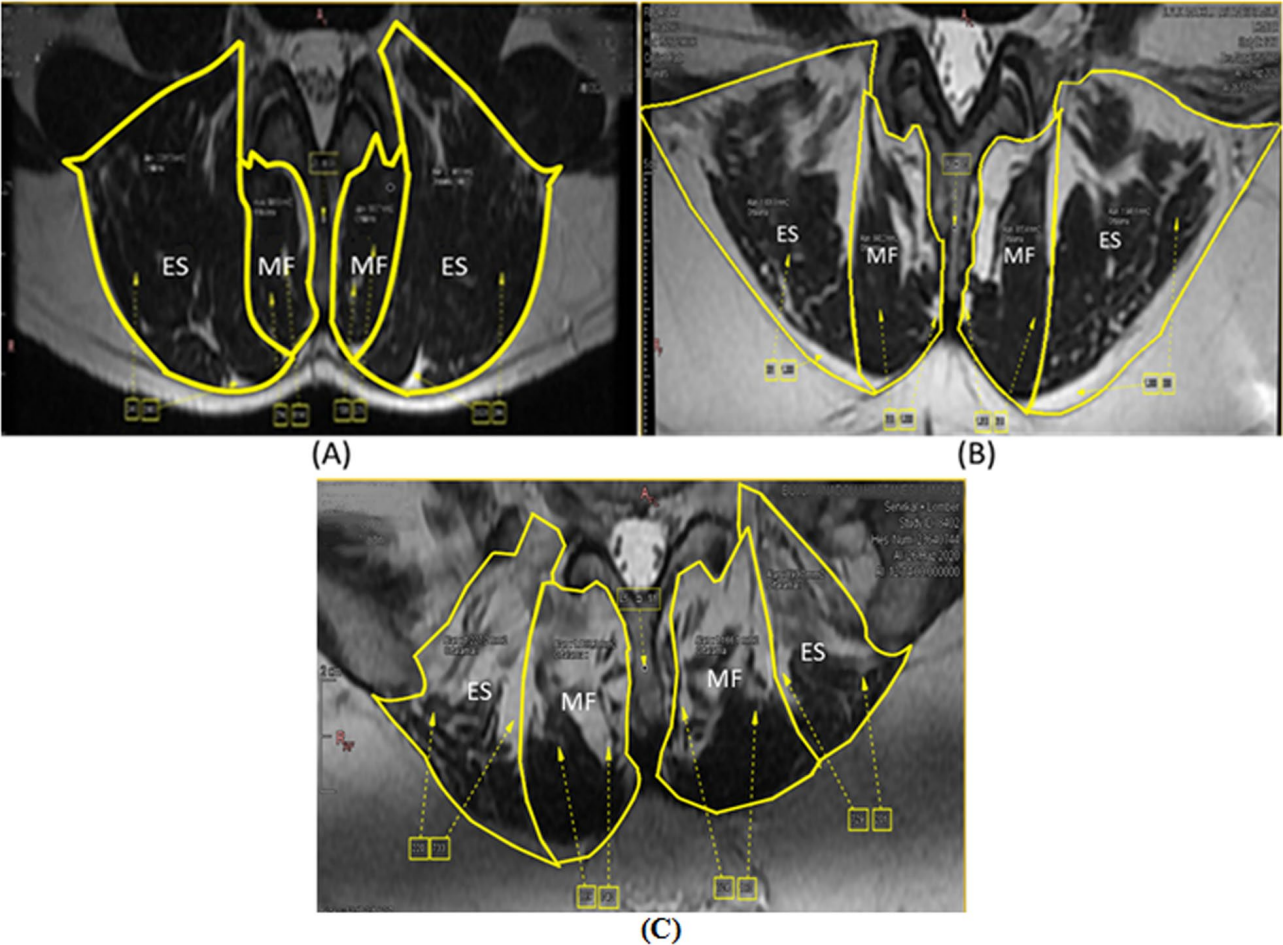
Degrees of fat infiltration; MF: musculus multifidus, ES: musculus erector spinae and **A** Grade 1: < 10% fat infiltration **B** Grade 2: 10–50% fat infiltration **C** Grade 3: > 50% fat infiltration

In this study, fat infiltration was evaluated semiquantitatively. The CSA was measured by determining the fascial borders (epimysium) of the muscles and using the PACS (picture archive and communication system) report and manual drawing. In the evaluation, fat infiltration was defined as the replacement of the muscle mass with fat and fibrous tissue, with < 10% as normal, 10%–50% as moderate, and > 50% as severe (Fig. 2) [26, 31]. Asymmetry was calculated as “the largest edge—the smallest edge/the largest edge × 100” and presented as a percentage (%) [67].

For intra- and interobserver reliability, 15 randomly selected patients were evaluated again after 1 month by the same radiologist (K.T.) and by a different radiologist (H.E.). The intraobserver kappa agreement value obtained was 0.953, and the interobserver agreement kappa value obtained was 0.935.

### Statistical analysis

Data obtained in the study were analysed statistically using IBM SPSS vn. 23 software (IBM, Chicago, IL, USA). Taking the CSA mean values into consideration, it was determined to be necessary to have a minimum sample size of 110 participants with 55 in each group for 95% confidence (1-α), 95% test power (1-β), and an effect size of f = 0.634. The study was completed with a total of 107 patients, 56 in the Non-rad group and 51 in the Rad group. According to the post hoc power analysis, the test power with 107 participants was determined to be 94.7% [68].

MANOVA was used to compare CSA and TCSA values according to group, segment, and sex. For multiple comparisons, the Duncan test was used. In the comparisons of age, BMI, the duration of low back pain, and VAS scores between the Rad and Non-rad groups, the Mann‒Whitney U test was used. The chi-square test was applied in the comparisons of categorical data. A value of *p* < 0.05 was accepted as statistically significant.

## Results

The demographic characteristics of the patients are given in Table 1. No significant differences were seen between the groups with respect to the mean values of age, sex, BMI, and the duration of low back pain. Statistically significant differences were determined between the groups with respect to pain severity as resting and activity VAS scores (*p* < 0.001).

**Table 1:** Comparisons of the demographic characteristics of the groups

	Non-rad		Rad		Total		*p*
	$\overline{\mathrm{x} }$± σ	Median (min–max)	$\overline{\mathrm{x} }$± σ	Median (min–max)	$\overline{\mathrm{x} }$± σ	Median (min–max)	
Age (years)	49.3 ± 9.2	49.5 (27–65)	49.5 ± 10.6	48 (23–65)	49.4 ± 9.8	49 (23–65)	0.931^1^
BMI (m^2^/kg)	29.3 ± 5	28.6 (20–43)	29.9 ± 5.3	29.8 (18–47)	29.6 ± 5.1	29.7 (18–47)	0.595^1^
LBP duration (months)	55.5 ± 55	36 (3–240)	53.2 ± 64.3	24 (3–300)	54.4 ± 59.3	36 (3–300)	0.604^2^
VAS resting	2.6 ± 1.5	2 (1–7)	5.9 ± 2.9	7 (1–10)	4.2 ± 2.8	3 (1–10)	< 0.001^2^
VAS activity	4.7 ± 1.9	4.5 (2–9)	7.8 ± 2.4	8 (2–10)	6.2 ± 2.6	6 (2–10)	< 0.001^2^
Sex	n (%)		n (%)				
Male	13 (23.2)		17 (33.3)		30 (28.0)		0.343^3^
Female	43 (76.8)		34 (66.7)		77 (72.0)		

^1^Independent sample t test, ^2^Mann‒Whitney U test, ^3^ Pearson chi-squared test, LBP: low back pain

The comparisons of the CSA and TCSA values of the groups according to sex and segment are presented in Table 2. No difference was seen in the CSA of the right and left MF and the left ES between the groups, but a significant difference was seen in the right ES (*p* = 0.021). The mean CSA value of the Non-rad group was lower. A significant difference was seen in the mean right TCSA values between groups, with lower values obtained in the Non-rad group (*p* = 0.005).

**Table 2:** Comparisons of the CSA and TCSA values of the groups according to sex and segment

	Group		Sex		Segment		Group*Sex		Sex*Segment		Group*Segment		Group*Sex*Segment	
	*p*	${\upeta }^{2}$	*p*	${\upeta }^{2}$	*p*	${\upeta }^{2}$	*p*	${\upeta }^{2}$	*p*	${\upeta }^{2}$	*p*	${\upeta }^{2}$	*p*	${\upeta }^{2}$
Right MF (CSA, mm^2^)	0.075	0.010	0.000	0.051	0.000	0.533	0.319	0.003	0.998	0.000	0.857	0.001	0.609	0.003
Left MF (CSA, mm^2^)	0.579	0.001	0.640	0.001	0.000	0.103	0.718	0.000	0.618	0.003	0.731	0.002	0.759	0.002
Right ES (CSA, mm^2^)	0.021	0.017	0.000	0.100	0.000	0.286	0.245	0.004	0.111	0.014	0.005	0.034	0.980	0.000
Left ES (CSA, mm^2^)	0.059	0.012	0.000	0.063	0.000	0.261	0.022	0.017	0.307	0.008	0.043	0.020	0.879	0.001
Right TCSA (Right MF + Right ES, mm^2^)	0.005	0.025	0.000	0.133	0.000	0.051	0.152	0.007	0.159	0.012	0.013	0.028	0.979	0.000
Left TCSA (Left MF + Left ES, mm^2^)	0.518	0.001	0.003	0.029	0.193	0.011	0.298	0.004	0.247	0.009	0.691	0.002	0.781	0.002

${\upeta }^{2}$: Partial eta squared, age and BMI were added to the model as covariate variables. MF: lumbar musculus multifidus, ES: lumbar musculus erector spinae

A significant difference was seen in the right MF, right and left ES mean CSA values between groups, and a difference was seen in the right and left mean TCSA values according to sex, with higher values obtained in males than in females (*p* < 0.001, *p* < 0.001, *p* < 0.001, *p* < 0.001, and *p* = 0.003, respectively).

According to segments, the highest mean values for the right and left MF CSA were seen in the L5-S1 segment and the lowest mean values were seen in the L3-L4 segment (*p* < 0.001). The highest mean values for the right and left ES CSA were seen in the L3-L4 segment, and the lowest mean values were seen in the L5-S1 segment (*p* < 0.001). A significant difference was also seen in the mean TCSA values according to segments (*p* < 0.001).

No difference was observed between the mean values according to sex or segment. The mean right and left ES CSA values in the L5-S1 segment according to group and segment were determined to be lower in the Rad group than in the Non-rad group (*p* = 0.005, *p* = 0.043).

The results of the correlation analyses between the CSA values of the groups and age, BMI, pain duration, and pain severity (resting and activity VAS) scores are presented in Table 3. In the Non-rad group, a weak positive correlation was found between age and the left ES CSA and left TCSA (r = 0.270 and r = 0.271, respectively). In the Rad group, a weak negative correlation was found between age and the right MF CSA (r = − 0.281). In the Non-rad group, a moderate-level positive correlation was found between BMI and the right and left ES CSA and TCSA (r = 0.520, r = 0.482, r = 0.462, r = 0.422, respectively). No relationship was found between the duration and severity of pain and the CSA or TCSA.

**Table 3:** Results of the correlation analyses between the CSA values of the groups and clinical features

Group		Age	BMI	Duration of LBP	VAS resting	VAS activity
Non-rad	Right MF (CSA, mm^2^)	0.104	0.023	− 0.031	− 0.219	− 0.055
	Left MF (CSA, mm^2^)	0.089	− 0.024	− 0.093	− 0.181	− 0.002
	Right ES (CSA, mm^2^)	0.174	0.520^*^	− 0.059	− 0.119	− 0.022
	Left ES (CSA, mm^2^)	0.270^*^	0.482^*^	− 0.093	− 0.052	0.062
	Right TCSA (Right MF + Right ES, mm^2^)	0.184	0.462^*^	− 0.061	− 0.170	− 0.036
	Left TCSA (Left MF + Left ES, mm^2^)	0.271^*^	0.422^*^	− 0.114	− 0.108	0.055
Rad	Right MF (CSA, mm^2^)	− 0.281^*^	0.120	− 0.165	− 0.093	− 0.138
	Left MF (CSA, mm^2^)	− 0.125	0.220	− 0.007	− 0.089	− 0.051
	Right ES (CSA, mm^2^)	− 0.027	0.088	− 0.073	0.183	0.133
	Left ES (CSA, mm^2^)	0.016	− 0.004	− 0.074	0.270	0.208
	Right TCSA (Right MF + Right ES, mm^2^)	− 0.110	0.116	− 0.116	0.135	0.076
	Left TCSA (Left MF + Left ES, mm^2^)	− 0.032	0.079	− 0.070	0.211	0.169

*Significant correlation at the level of 1%, MF: lumbar musculus multifidus, ES: lumbar musculus erector spinae, VAS: visual analogue scale, LBP: low back pain

The fat infiltration values according to group are presented in Table 4. A significant difference was seen between the groups in the fat infiltration of the right MF (*p* = 0.015). Grade 2 fat infiltration (10–50%) was found in 72% of the Non-rad group and in 56.9% of the Rad group. Grade 3 fat infiltration (> 50%) was seen at a higher rate in the right and left MF and left ES in the Rad group than in the Non-rad group.

**Table 4:** Comparisons of the fat infiltration values of the MF and ES of the groups

	Non-rad	Rad	Total	*p*
*Fat Infiltration_Right MF*				
< 10%	36 (21.4)	47 (30.7)	83 (25.9)	0.015
10%-50%	121 (72)^a^	87 (56.9)^b^	208 (64.8)	
> 50%	11 (6.5)^a^	19 (12.4)^b^	30 (9.3)	
*Fat Infiltration_Left MF*				
< 10%	43 (25.6)	42 (27.5)	85 (26.5)	0.088
10%-50%	116 (69)	93 (60.8)	209 (65.1)	
> 50%	9 (5.4)	18 (11.8)	27 (8.4)	
*Fat Infiltration_Right ES*				
< 10%	40 (23.8)	31 (20.3)	71 (22.1)	0.679
10%-50%	111 (66.1)	108 (70.6)	219 (68.2)	
> 50%	17 (10.1)	14 (9.2)	31 (9.7)	
*Fat Infiltration_Left ES*				
< 10%	41 (24.4)	33 (21.6)	74 (23.1)	0.297
10%-50%	118 (70.2)	105 (68.6)	223 (69.5)	
> 50%	9 (5.4)	15 (9.8)	24 (7.5)	

^a^^−^^a^No difference between groups with the same letter, ^a−b^A difference was found between groups with different letters

A significant difference was found in the right and left MF fat infiltration according to age, BMI, and pain duration (*p* < 0.001, *p* < 0.001, *p* = 0.004, *p* < 0.001, *p* < 0.001, *p* = 0.003, respectively), but no difference was determined according to VAS activity or VAS resting scores (*p* = 0.129, *p* = 0.062, *p* = 0.423, *p* = 0.300, respectively).

A significant difference was found in the right and left ES fat infiltration according to age, BMI, and pain duration (*p* < 0.001, *p* < 0.001, *p* < 0.001, respectively), but no difference was found according to VAS activity or VAS resting scores (*p* = 0.251, *p* = 0.359, respectively).

The comparisons of categorical data according to fat infiltration are presented in Table 5. No difference in fat infiltration was seen according to herniation level or radiculopathy side. When examined according to segments, in the right and left MF and ES, the rate of Grade 1 (< 10%) fat infiltration was higher in the L3-L4 segment, and the rate of Grade 2 (10%-50%) fat infiltration was higher in the L4-L5 and L5-S1 segments. When examined according to sex, a significantly higher rate of fat infiltration was seen in females than in males (*p* < 0.001).

**Table 5:** Comparisons of categorical data according to fat infiltration

	Hernia level			Radiculopathy side			Segment			Sex	
	L3-L4	L4-L5	L5-S1	Bilateral	Right	Left	L3-L4	L4-L5	L5-S1	Male	Female
*Right MF*											
< 10%	23 (41.1)	40 (44)	39 (51.3)	6 (54.5)	8 (47.1)	13 (56.5)	52 (48.6)^a^	20 (18.7)^b^	11 (10.3)^b^	44 (48.9)^a^	39 (16.9)^b^
10%-50%	29 (51.8)	47 (51.6)	33 (43.4)	4 (36.4)	8 (47.1)	10 (43.5)	51 (47.7)^a^	79 (73.8)^b^	78 (72.9)^b^	45 (50)^a^	163 (70.6)^b^
> 50%	4 (7.1)	4 (4.4)	4 (5.3)	1 (9.1)	1 (5.9)	0 (0)	4 (3.7)^a^	8 (7.5)^ab^	18 (16.8)^b^	1 (1.1)^a^	29 (12.6)^b^
*Left MF*											
< 10%	24 (42.9)	43 (47.3)	39 (51.3)	4 (36.4)	8 (47.1)	13 (56.5)	55 (51.4)^a^	21 (19.6)^b^	9 (8.4)^b^	44 (48.9)^a^	41 (17.7)^b^
10%-50%	27 (48.2)	43 (47.3)	33 (43.4)	6 (54.5)	7 (41.2)	10 (43.5)	47 (43.9)^a^	79 (73.8)^b^	83 (77.6)^b^	45 (50)^a^	164 (71)^b^
> 50%	5 (8.9)	5 (5.5)	4 (5.3)	1 (9.1)	2 (11.8)	0 (0)	5 (4.7)	7 (6.5)	15 (14)	1 (1.1)^a^	26 (11.3)^b^
*Right ES*											
< 10%	17 (30.4)	34 (37.4)	32 (42.1)	3 (27.3)	5 (29.4)	11 (47.8)	47 (43.9)^a^	18 (16.8)^b^	6 (5.6)^c^	33 (36.7)^a^	38 (16.5)^b^
10%-50%	38 (67.9)	56 (61.5)	43 (56.6)	8 (72.7)	12 (70.6)	12 (52.2)	59 (55.1)^a^	83 (77.6)^b^	77 (72)^b^	56 (62.2)^a^	163 (70.6)^a^
> 50%	1 (1.8)	1 (1.1)	1 (1.3)				1 (0.9)^a^	6 (5.6)^a^	24 (22.4)^b^	1 (1.1)^a^	30 (13)^b^
*Left ES*											
< 10%	19 (33.9)	37 (40.7)	33 (43.4)	3 (27.3)	5 (29.4)	11 (47.8)	50 (46.7)^a^	19 (17.8)^b^	5 (4.7)^c^	38 (42.2)^a^	36 (15.6)^b^
10%-50%	37 (66.1)	54 (59.3)	43 (56.6)	8 (72.7)	12 (70.6)	12 (52.2)	57 (53.3)^a^	84 (78.5)^b^	82 (76.6)^b^	49 (54.4)^a^	174 (75.3)^b^
> 50%	0 (0)	0 (0)	0 (0)				0 (0)^a^	4 (3.7)^a^	20 (18.7)^b^	3 (3.3)^a^	21 (9.1)^b^

^a^^−^^a, b^^−^^b^No difference between groups with the same letter, ^a−b−c^A difference was found between groups with different letters

The distribution of fat infiltration in the MF and ES according to the number of herniations is presented in Table 6. A significant difference was seen in the right and left ES fat infiltration distributions (*p* < 0.001). A difference was seen in the distribution between patients with one herniation and those with three herniations. The rate of Grade 1 (< 10%) fat infiltration was higher in patients with a single herniation, and as the number of herniations increased, the rate and severity of fat infiltration increased.

**Table 6:** Distribution of fat infiltration in the MF and ES according to the number of herniations

	Number of herniations		
	1	2	3
*Right MF*			
< 10%	18 (56.3)	18 (52.9)	16 (39)
10–50%	14 (43.8)	16 (47.1)	21 (51.2)
> 50%	0 (0)	0 (0)	4 (9.8)
*Left MF*			
< 10%	20 (62.5)	19 (55.9)	16 (39)
10–50%	12 (37.5)	14 (41.2)	21 (51.2)
> 50%	0 (0)	1 (2.9)	4 (9.8)
*Right ES*			
< 10%	21 (65.6)^a^	16 (47.1)^ab^	10 (24.4)^b^
10–50%	11 (34.4)^a^	18 (52.9)^ab^	30 (73.2)^b^
> 50%	0 (0)	0 (0)	1 (2.4)
*Left ES*			
< 10%	22 (68.8)^a^	17 (50)^ab^	11 (26.8)^b^
10–50%	10 (31.3)^a^	17 (50)^ab^	30 (73.2)^b^
> 50%	–	–	–

^a^^−^^a, b^^−^^b, ab^^−^^ab^No difference between groups with the same letter, ^a−b^A difference was found between groups with different letters, ^ab^There was no difference from the other two groups

The comparisons of the presence of asymmetry according to group are presented in Table 7. No difference was found between the groups with respect to MF and ES asymmetry. No difference was found between the groups with respect to TCSA asymmetry.

**Table 7:** Comparisons of the presence of asymmetry according to group

	Non-rad	Rad	Total	*p*
MF	60 (35.7)	58 (37.9)	118 (36.8)	0.684
ES	64 (38.1)	59 (38.6)	123 (38.3)	0.932
TCSA	36 (21.4)	35 (22.9)	71 (22.1)	0.755

MF: lumbar musculus multifidus, ES: lumbar musculus erector spinae, TCSA: total cross-sectional area, Non-rad: lumbar disc herniation group without radiculopathy, Rad: lumbar disc herniation group with radiculopathy

When the general levels of herniation were examined, in the Non-rad group, bilateral herniation was found in 85.7%, right-side herniation in 3.6%, and left-side herniation in 10.7% of patients. In the Rad group, bilateral herniation was found in 63.5%, right-side herniation in 38.5%, and left-side herniation in 61.5% of patients.

A difference in the distribution of herniation levels according to group was observed (*p* = 0.087). The distribution of levels of herniation was 46.4% in the L3-L4 segment, 88.1% in the L4-L5 segment, and 62.5% in the L5-S1 segment in the Non-rad group and 58.8% in the L3-L4 segment, 88.2% in the L4-L5 segment, and 80.4% in the L5-S1 segment in the Rad group (Table 8).

**Table 8:** Comparisons of herniation levels according to group

	Non-rad	Rad	P*
L3-L4	26 (46.4)	30 (58.8)	0.087
L4-L5	46 (88.1)	45 (88.2)	
L5-S1	35 (62.5)	41 (80.4)	

*Chi-squared test, n (%)

## Discussion

In the results of this study, no significant difference was seen in the CSA values between the groups. Only the mean CSA value in the right ES was lower in the Non-rad group, and similarly, the mean right TCSA values were lower. In a study by Hyun et al. [69] of groups with disc herniation with and without radiculopathy, no significant difference was observed between the sides with respect to the TCSA or functional CSA (FCSA), but the FCSA values were higher in the group without radiculopathy than in the group with radiculopathy. In another study of patients operated on because of LDH, no difference was seen in the paraspinal muscle CSA values compared with the less affected side [70]. Some studies have reported the hypertrophy of the MF according to the CSA in LDH patients compared to control participants [34, 71]. In the current study, the right ES CSA and right TCSA values of the Non-rad group were lower than those of the Rad group, suggesting that increased CSA (pseudohypertrophy) was associated with severe fat infiltration of > 50% in the Rad group. Despite a decrease in FCSA when there is pseudohypertrophy, an increase is observed in TCSA because of the increasing fat tissue [25, 26]. The findings determined in the current study support this view.

In the current study, Grade 3 fat infiltration (> 50%) was found at a higher rate in the Rad group than in the Non-rad group. Ji Hye Min et al. [30] reported a relationship between radiculopathy and fat infiltration in the MF muscle associated with more severe and widespread atrophy. Kader et al. [72] determined that MF atrophy was associated with fat infiltration in 80% of patients with disc degeneration and nerve compression identified on MRI scans and showed that there was more severe and widespread MF atrophy in patients with radiculopathy than in those without radiculopathy. Chen et al. [23] showed that in patients with radiculopathy treated with microdiscectomy, markers for impaired muscle regeneration were associated with worse outcomes. In an experimental study of disc injury, Hodge et al. [73, 74] reported that after 6 months, there was an increase histologically in fat and connective tissue in the MF, but atrophy was not identified, and these findings were stated to be compatible with gene expression data from cytokines that play a role in both adipogenesis and fibrosis. It has been suggested that fat infiltration in muscles mediated by proinflammatory cytokines and muscle inhibition in the acute and subacute phases is associated with nonuse in later stages [24]. Similar to these findings in the literature, grade 2 and 3 fat infiltration and associated muscle atrophy were found in 73.3%–77.9% of the patients in both groups of the current study [53, 72]. As in other studies, atrophy associated with fat infiltration was evident, especially at the L5-S1 level [26, 30, 72]. As the number of herniations increased, the ratio of the involvement of the right–left ES muscles and the severity of fat infiltration significantly increased. Although the levels of fat infiltration seemed to be similar between the Rad and Non-rad groups, a higher rate of severe (> 50%) fat infiltration was observed in the Rad group. This suggests that nerve root compression originates from the increased severity of degeneration in the paraspinal muscles. When intervertebral foramens are under pressure (disc-related nerve root compression, spinal stenosis), denervation may explain fat infiltration and atrophy [75]. The results of the current study were consistent with findings in the literature.

No difference was found between the two groups with respect to asymmetry in the current study. Previous studies of healthy individuals have reported differences of 3 ± 4% [76] and 7.2 ± 9.6% [77] between the right and left sides compared to the largest edge. Based on these results, it has been reported that a difference of > 10% can be defined as asymmetry [26]. Kader et al. [72] stated that the majority of muscle degeneration in patients with unilateral radiculopathy at a single level was bilateral and multilevel. Ji Hye Min et al. [30] found no difference in MF asymmetry between bilateral and unilateral nerve root compression and bilateral MF atrophy in most patients with unilateral radiculopathy. In a study by Fortin et al. [35], there was no significant MF asymmetry at the upper or lower spinal segments of the level of disc herniation. The results of the current study showed that muscle degeneration not only occurred on the side of disc herniation and nerve compression but also developed on the contralateral side and/or in the upper and lower segments. In addition, it was thought that there was no difference between the groups with respect to asymmetry because muscle atrophy and fat infiltration were distributed equally on the right and left sides. Thus, it can be concluded that in patients with LDH with and without nerve compression, muscle degeneration developed after 3 months regardless of the herniation side, and as observed in patients with acute unilateral LDH [27], degeneration is not localised only on the side of the herniated disc but is widespread, involving the opposite segment and/or upper and lower segments. The current study results were consistent with the literature. However, in some studies, a relationship between paraspinal muscle asymmetry and unilateral low back pain with or without radiculopathy has been observed [31, 33, 36, 38, 76, 78, 79].

In the current study, the CSA and TCSA values of males were found to be higher than those of females. Previous studies have also shown a greater CSA and higher density of paraspinal muscles in males than in females [18, 26, 30, 72, 80, 81]. The current study results were consistent with the literature.

The results of the current study demonstrated a weak and moderate-level relationship between the CSA and TCSA values of the muscles and age and BMI. Studies in the literature have reported different results related to age. Some studies have stated that the CSA of muscles decreases with age [26, 30, 72, 81, 82], while others have shown no such relationship [68, 80, 83, 84]. Some studies have found a significant correlation between BMI and muscle values and have stated that paraspinal muscle changes are related to BMI [85], whereas others have stated that there is no relationship between BMI and CSA [53, 68, 82].

In the current study, age, sex, BMI, and the duration of pain were found to be related to fat infiltration. A higher rate of fat infiltration was found in females than in males. An increase in fat infiltration with ageing was seen in both groups, and as BMI increased, there was an increase in fat infiltration. As the duration of pain increased, the rate of fat infiltration increased in both groups. Fat infiltration of the paraspinal muscles was generally observed in the area adjacent to the vertebra. In the literature, a higher rate of fat infiltration in females than in males has been reported [53, 86, 87]. Kjaer et al. [53] suggested that the significant difference in fat infiltration in the MF muscles of males and females could be a result of differences in body composition, and the higher rate of fat infiltration in the MF in females could be a reflection of the higher percentage of body fat in females. Studies in the literature have stated that fat infiltration increases with ageing [86, 88–90]. Mcloughlin et al. [89] determined a relationship between paraspinal fat accumulation and age and the amount of subcutaneous fat and stated that this was a sign of paraspinal muscle atrophy in patients who had not undergone surgery. Some studies have shown a relationship between BMI and fat infiltration [21, 83], while others have found no relationship [53, 82]. In three studies by Ranger et al., which examined the relationship between MF fat infiltration and the duration of pain, there was limited evidence of no significant relationship between periods of less than or more than one year and fat infiltration [32]. According to the results of another study, there was a relationship between the duration of pain and the severity of MF muscle atrophy, although not at a statistically significant level, and it was stated that high pain scores tended to indicate an increase in muscle atrophy [26].

In the current study, fat infiltration was determined to be the lowest at L3-L4 and the greatest at L5-S1. According to the group–segment interaction, the right ES CSA was lowest in the L5-S1 segment in the Rad group, which was lower than that in the Non-rad group. The involvement of the MF was greater at L4-L5 and L5-S1, and in the ES, it was greater at L5-S1. The VAS resting and activity scores were found to be higher in the Rad group with severe (> 50%) fat infiltration than in the Non-rad group. In a recent study, it was reported that there was greater fat infiltration in the multifidus at the L4-L5 level in patients with a higher VAS score than in those with a lower VAS score, and to compensate for this, less fat infiltration formed in the psoas [16].

Muscles are tissues with the capacity to renew. Under various stimuli (neural activation, denervation, wounds and an inflammatory environment, exercise, flexion, and overloading), changes may occur in muscle measurements, and these changes affect the muscle strength capability [91]. In individuals with regular physical activity, the proinflammatory response against intervertebral disc degeneration is reduced, fibrosis is reduced, and through the regulation of the gene network in the MF, most harmful effects are prevented [39]. To reverse, reduce, or halt the degenerative changes in the paraspinal muscles, which are related to LDH, it is important that pharmacological treatment and appropriate exercise programmes determined according to the psychological and social status of the patient are prescribed and that patients visiting the clinic are informed about and referred to these exercise programmes.

A limitation of the current study was that there was no control group. Therefore, no comparison could be made with the fat infiltration level and asymmetry of healthy individuals. However, previous studies of healthy individuals have reported a relationship between fat infiltration and LDH and that there is asymmetry of > 10% difference between the right and left sides [20, 26, 30, 35]. Other limitations were that there were no evaluations of all the lumbar paraspinal muscles at all lumbar levels (L1-S1), disc degeneration, endplate changes (Modic changes), or facet joint degeneration [16, 92–95]. The facts that the patients were not classified according to decade age groups and the subcutaneous fat index was not used instead of BMI are other limitations of our study [96, 97].

## Conclusion

In conclusion, there seems to be a relationship between widespread (2 or more lumbar segments) involvement of the lumbar paraspinal muscles together with severe (> 50%) fat infiltration and radiculopathy. Denervation in disc herniations, reinnervation, and nonuse are said to lead to atrophy in paraspinal muscles and increased fat infiltration in the muscles [98, 99]. By becoming more evident in the presence of root compression, this seems to cause more severe degeneration in the muscles and fat infiltration. This result shows the need for immediate correction of root compression in patients with radiculopathy, first with appropriate medical treatment methods (anti-inflammatory drugs, muscle relaxants, opioids when necessary, pain control with local applications and/or intense physiotherapy (massage, ultrasound, electric stimulation, exercises, magnets, manipulation) and then, if these are not successful, appropriate surgical methods to prevent the progression of denervation.

## Data Availability

The data obtained and analysed in this study are not available to the public because of ethical regulations and local management procedures but can be obtained on request from the corresponding author.

## Abbreviations

MF: Lumbar musculus multifidus
ES: Lumbar musculus erector spinae
MRI: Magnetic resonance imaging
CSA: Cross-sectional area
TCSA: Total cross-sectional area
FCSA: Functional cross-sectional area
VAS: Visual analogue scale
LDH: Lumbar disc herniation
BMI: Body mass index
EMG: Electromyography
CT: Computed tomography
Non-rad: Lumbar disc herniation group without radiculopathy
Rad: Lumbar disc herniation group with radiculopathy
